# The centenary of Hereditas - almost a fairytale

**DOI:** 10.1186/s41065-020-00156-8

**Published:** 2020-11-07

**Authors:** Yongyong Shi, Stefan Baumgartner

**Affiliations:** 1 Bio-X Institutes, Shanghai Jiao Tong University, Shanghai, 20030 China; 2 Department of Experimental Medical Sciences, Lund University, 22100 Lund, Sweden

## Abstract

This is the Editorial for a series of articles illuminating the history and success story of Hereditas, one of the oldest journals in the area of genetics. For this reason, we invite you to read this special series on a journal with a distinct reputation and which can celebrate its Centenary in 2020.

## Background

The fairytale of Hereditas began in 1920 when a group of geneticists, the previous founders of the Mendelian Society in Lund in 1910 decided that they needed a platform to be able to publish scientific articles and to reach out to colleagues and scientists outside of Sweden. This led to the establishment of Hereditas in 1920. The sequence of events is meticulously narrated by [1]. Two protagonists promoting the foundation of Hereditas should be named here: Herman Nilsson-Ehle (1873–1949) and Robert Larsson (1885–1956). The latter also became the first Editor-in-Chief (EiC) holding this post for more than 3 decades from 1920 to 1954, followed by Arne Müntzing (1955–1977), Arne Lundqvist (1978–1988), Karl Fredga and Arne Lundqvist (1989–1996), Ulf Gyllensten (1996–2001), Anssi Saura (2002–2011), Stefan Baumgartner (2012 -), and Yongyong Shi (2016 -, in parallel to Baumgartner).

**Fig. 1:**
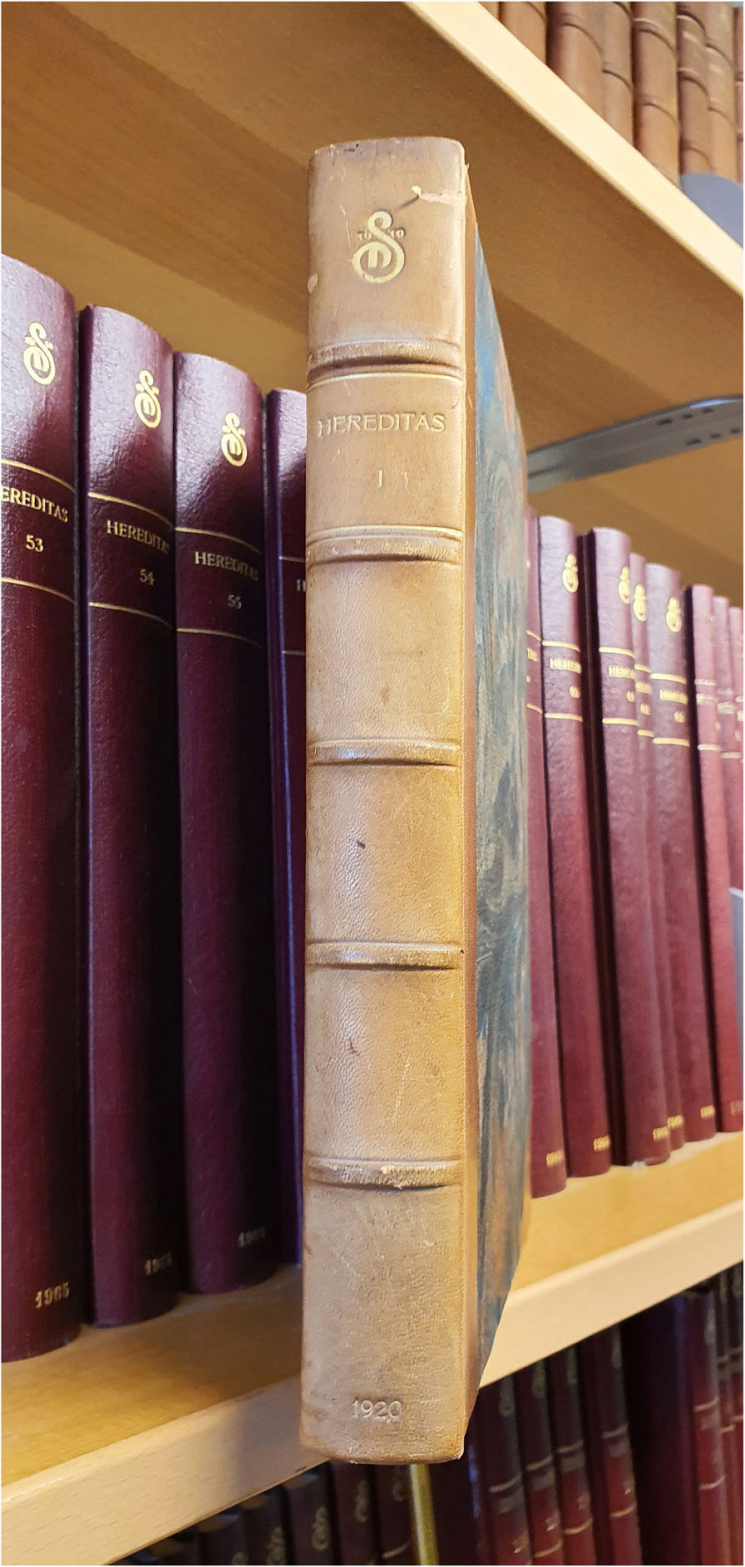
The “holy grail” of Hereditas: volume 1 from 1920 along with the whole collection of back issues. The collection is located in Lund (Sweden). Note the sign of the Mendelian Society of Lund on the top of the book spine

In 2005, an important decision was taken by the EiC at the time, Anssi Saura, to change the subscription format of Hereditas to open-access (OA), thus helping to pioneer the path of OA-journals. This led to an increase of submissions and provided immediate access to Hereditas to all scientists world-wide. Another wise decision was taken in 2011 when the owner of Hereditas, the Mendelian Society of Lund, sponsored by the Erik Philip-Sörensen Foundation, decided to digitalize all back-issues from the very first one in 1920 (Fig. 1). It was an extraordinarily important decision to ensure that all back-issues were available to the scientific readership. In 2013 and 2014, the journal experienced a dramatic drop of submissions which forced the EiC to terminate its activity at the end of 2014. In an astonishing turn of events, a few months later, the journal experienced a resurrection with the collaboration with a new publisher, Springer Nature which in retrospect became an extremely beneficial union for both sides.

We have divided this Centenary issue into topical areas. These sections represent prominently covered topics by Hereditas over the last 100 years: cytogenetics, race biology, crop genetics and evolution. The topical series will be complemented by a bibliometric overview on all articles published by Hereditas. Some of the articles proved to be “high flyers” with a high number of citations. Of note is a report in the area of cytogenetics on a proposal for a common nomenclature for centromeric positions on chromosomes, published in 1964 [2]. This publication was cited about 8000 times (source: Google Science). No lesser attention should to be given to an article that presented for the first time the number of chromosomes in humans [3]. From today’s perspective, it is inconceivable that it took such an extraordinary amount of time for the scientific community to accept that humans have 46 chromosomes. The laboratory in Lund where this seminal publication originated from is commemorated by a metal plate. The story and credits for this discovery is also illuminated in by an article of this topical series.

The Centenary year 2020 also experienced a dramatic increase of the impact factor (IF) to a whopping 2.4. This figure is a clear sign that scientists regard Hereditas as a valid platform to publish their data. Consequently, a record number of submissions were received, exceeding the number in 2019 by a factor of 2. While this increased number of submissions was encouraging, the EiCs decided this year to raise the quality threshold for incoming manuscripts to avoid another increase of manuscripts.

## Conclusions

The current EiCs wish to thank all past scientific members of Hereditas, be it the previous EiCs, subject Editors or Technical Editors for their significant contributions towards the success of this journal. We also wish to thank former and present publishers, Wiley and Springer Nature for their benevolence. Without all the above-mentioned actors, the story of Hereditas would probably have been narrated to future generations as a fairytale, simply because how many scientific journals can boast the celebration of a Centenary?

## Data Availability

The datasets generated during and/or analyzed during the current study are available from the corresponding author on reasonable request.
